# Supplementary material for: AP2 hemicomplexes contribute independently to synaptic vesicle endocytosis
Source: eLife. 2013 Mar 5;2:e00190. doi: 10.7554/eLife.00190 (PMC3591783; doi:10.7554/eLife.00190)
Supplement: Supplementary file 1. — Strains. DOI: http://dx.doi.org/10.7554/eLife.00190.025 [file elife-00190-supp1.doc]

**Supplementary file 1: Strains**

| APA-2 translational GFP | |
| --- | --- |
|  | EG4521 *lin-15(n765ts)X; oxEx947[apa-2::GFP; lin-15(+)]* |
|  | EG7496 *apa-2(b1044) X ; oxEx1122[apa-2::GFP,cc::GFP]* |
|  | EG7497 *apa-2(ox422) X ; oxEx1122[apa-2::GFP,cc::GFP]* |
|  | |
| CD4 endocytosis assay | |
|  | EG7043 *oxSi482[*P*vha-6::eGF*P*::CD4-LL(di-leucine); unc-119(+)] II; unc-119(ed3) III* |
|  | EG7044 *oxSi482[*P*vha-6::eGFP::CD4-LL(di-leucine); unc-119(+)] II; apa-2(ox422) X*  EG7720 *oxSi482[Pvha-6::eGFP::CD4-LL(di-leucine); unc-119(+)] II; apm-2(e840) X* |
|  | |
| yolk-uptake assay | |
|  | RT130 *pwIs23[vit-2::GFP]* |
|  | EG7045 *pwIs23[vit-2::GFP]; apa-2(ox422) X* |
|  | EG7046 *pwIs23[vit-2::GFP]; apa-2(b1044) X* |
| APA-2::GFP localization  EG8012 *oxSi254[*P*dpy-30::apa-2::GFP; unc-119(+)] II; unc-119(ed3) III*  EG7763 *oxSi254[*P*dpy-30::apa-2::GFP; unc-119(+)] II; apm-2(e840) X* | |
| APM-2::GFP localization | |
|  | EG6291 *oxSi54[*P*dpy-30::apm-2::GFP; unc-119(+)] II; apm-2(e840) X* |
|  | EG6292 *oxSi54[*P*dpy-30::apm-2::GFP; unc-119(+)] II; apm-2(e840) apa-2(ox422) X* |
|  | |
| sigma2::GFP localization | |
|  | EG6293 *oxSi108[aps-2::GFP; unc-119(+)] II; unc-119(ed3) III* |
|  | EG6294 *oxSi108[aps-2::GFP; unc-119(+)] II; apm-2(e840) X* |
|  | EG6295 *oxSi108[aps-2::GFP; unc-119(+)] II; apa-2(ox422) X* |
|  | EG6296 *+/szT1[lon-2(e678)] I; oxSi108[aps-2::GFP; unc-119(+)] II; szT1/apm-2(e840) apa-2(ox422) X* |
|  | |
| beta localization | |
|  | EG6517 *oxSi381[*P*dpy-30::apb-1(cDNA)::GFP; unc-119(+)] II; unc-119(ed3) III* |
|  | EG7047 *oxSi381[*P*dpy-30::apb-1(cDNA)::GFP; unc-119(+)] II; apa-2(ox422) X* |
|  | EG7048 *oxSi381[*P*dpy-30::apb-1(cDNA)::GFP; unc-119(+)] II; apm-2(e840) X* |
|  | |
| APA-2 and SNB-1 colocalization | |
|  | EG6155 *dkIs160[*P*unc-25::GFP::apa-2; unc-119(+)]; oxEx1411[*P*unc-47::snb-1::tagRFP;* P*unc-122::GFP]* |
|  | |
| vesicle protein localization | |
|  | EG5932 *snt-1(md290) II; unc-119(ed3) III; oxSi180[snt-1::GFP; unc-119(+)] IV* |
|  | EG6156 *oxSi180[snt-1::GFP; unc-119(+)] IV; apa-2(ox422) X* |
|  | EG6157 *oxSi180[snt-1::GFP; unc-119(+)] IV; apa-2(b1044) X* |
|  | EG6159 *+/szT1[lon-2(e678)] I; oxSi180[snt-1::GFP; unc-119(+)] IV; szT1/apm-2(e840) apa-2(ox422) X* |
|  | EG5717 *unc-119(ed3) III; oxSi36[unc-47::GFP; unc-119(+) ] IV* |
|  | EG6160 *oxSi36[unc-47::GFP; unc-119(+)] IV; apa-2(ox422) X* |
|  | EG6161 *oxSi36[unc-47::GFP unc-119(+)] IV; apa-2(b1044) X* |
|  | EG6162 *+/szT1[lon-2(e678)] I; oxSi36[unc-47::GFP unc-119(+)] IV; szT1/apm-2(e840) apa-2(ox422) X* |
|  | EG6163 *unc-119(ed3) III; oxSi184[*P*unc-47::sng-1::GFP unc-119(+)] IV* |
|  | EG6164 *oxSi184[*P*unc-47::sng-1::GFP; unc-119(+)] IV; apa-2(ox422) X* |
|  | EG6165 *oxSi184[*P*unc-47::sng-1::GFP unc-119(+)] IV; apa-2(b1044) X* |
|  | EG6166 *+/szT1[lon-2(e678)] I; oxSi184[*P*unc-47::sng-1::GFP unc-119(+)] IV; szT1/apm-2(e840) apa-2(ox422) X* |
|  | |
| electron microscopy and electrophysiology | |
|  | EG6147 *apa-2(ox422) X* |
|  | EG6149 *oxSi254[*P*dpy-30::apa-2::GFP; unc-119(+)] II; apa-2(ox422) X* |
|  | EG6148 *oxSi253[*P*rab-3::apa-2::GFP, unc-119(+)] I; apa-2(ox422) X* |
|  | EG6150 *oxSi53[*P*dpy-7::apa-2::GFP unc-119(+)] I; apa-2(ox422) X* |
|  | EG6151 *apm-2(e840) apa-2(ox422) X; oxEx1452[*P*dpy-7::apa-2::mCherry;* P*dpy-7::apm-2::GFP;* P*unc-122::GFP]* |
